# D-Amino Acid Oxidase-pLG72 Interaction and D-Serine Modulation

**DOI:** 10.3389/fmolb.2018.00003

**Published:** 2018-01-24

**Authors:** Loredano Pollegioni, Luciano Piubelli, Gianluca Molla, Elena Rosini

**Affiliations:** ^1^Dipartimento di Biotecnologie e Scienze della Vita, Università degli Studi dell'Insubria, Varese, Italy; ^2^The Protein Factory, Politecnico di Milano and Università degli studi dell'Insubria, Milan, Italy

**Keywords:** D-amino acids, D-serine, flavoprotein, protein-protein interaction, schizophrenia

## Abstract

pLG72 is a small, primate-specific protein of 153 amino acids. It is the product of the *G72* gene, expressed in testis, spinal cord, and brain. The presence of *G72* transcript and pLG72 has recurrently been called into question, however *G72* mRNA and pLG72 protein levels were higher in blood and brain of patients with schizophrenia than in healthy controls. On the one hand, the SNP rs2391191 corresponding to the R30K substitution in pLG72 was genetically linked to schizophrenia, reduced thickness of the brain cortex in schizophrenia-affected individuals, and altered memory function. Various lines of evidence indicated that pLG72 is a mitochondrial protein, specifically an extrinsic protein bound on the outer membrane. Over the years, pLG72 was proposed to be involved in different functions: (a) overexpression induces mitochondria fragmentation, increasing the numbers of shorter and more mobile ones which could be delivered faster to regions of intense growth and facilitating the dendritic complexity; (b) it might induce oxidative stress by interacting with methionine-R-sulfoxide reductase B2; and (c) it binds and modulates the activity of FMN-containing oxidoreductase of the respiratory complex I. The main role of this protein, however, is related to its binding to the human flavoenzyme D-amino acid oxidase (hDAAO), i.e., the main catabolic enzyme for D-enantiomer of serine. This D-amino acid is a main endogenous coagonist of the N-methyl-D-aspartate type glutamate receptor (NMDAR) involved in main functions such as synaptic plasticity, learning, memory, and excitotoxicity. For this work, we reviewed the recent literature concerning the hDAAO-pLG72 interaction, focusing on the molecular details of the interaction, the effect of hDAAO function and stability, and the cellular effects, especially on D-serine concentration. The main effects related to the pathological R30K substitution are also reported. We have highlighted the gaps in our knowledge of this human protein as well as the relevance of clarifying the molecular details of hDAAO-pLG72 interaction in order to design molecules to modulate hDAAO activity/stability and thus NMDAR function acting at the D-serine cellular level.

## Introduction

**Figure 1:**
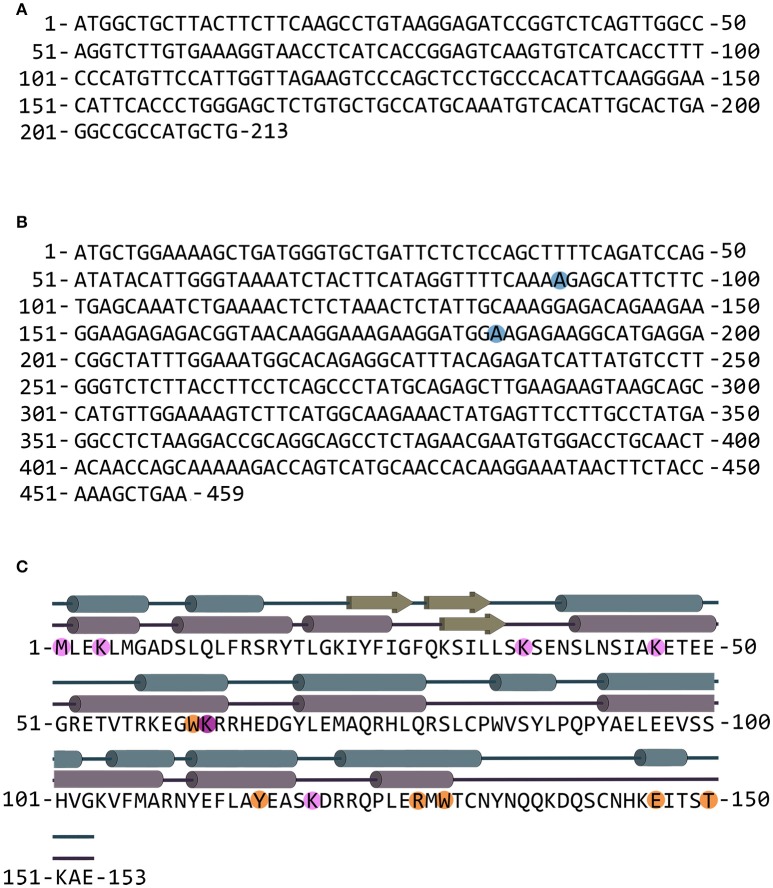
*G30* and *G72* genes and pLG72 protein. **(A)** Nucleotide sequence of the *G30* gene (genebank entry code AY138548.1); the gene is transcribed on the opposite DNA strand in comparison with *G72*. **(B)** Nucleotide sequence of the *G72* gene; blue circles indicate nucleotides that are different in SNPs rs2391191 (AGG→AAG resulting in the R→K substitution at position 30) and rs9558562 (AAG→GAG resulting in the K→E substitution at position 62). The reported sequence codes for K30/K62 pLG72 variant (genebank entry code NM_172370.4 with A at position 89). **(C)** Primary and secondary structure of pLG72; residues preceding the limited proteolysis sites are shown in orange and residues modified by BS^3^ cross-linking reagent are shown in pink. Cross-linked K62 residue is shown in purple (Birolo et al., 2016). The secondary structure is shown according to the final homology model (blue) and as predicted by PSSpred server (purple).

The *G72* and *G30* genes were first identified in 2002 on the long arm of chromosome 13 in a linkage study between schizophrenia and single nucleotide polymorphisms (SNPs) (Chumakov et al., 2002; Sacchi et al., 2016). Notably, no orthologs of *G72* gene are present in rodents or other species: it represents the rare case of a primate-specific gene. The longest ORF of the gene encodes for pLG72, a protein of 153 amino acids (RefSeq NM_172370, see Figure 1).

pLG72 isoform is expressed in testis, spinal cord, caudate nucleus, and amygdala libraries (Chumakov et al., 2002). In particular, pLG72 was identified in human prefrontal cortex, in primary astrocytes (Sacchi et al., 2008), and human amygdala and caudate nucleus (Kvajo et al., 2008). In transgenic mice carrying the entire human *G72* gene, the highest transcript level was apparent in the testis and in selected brain regions (Otte et al., 2009; Cheng et al., 2014). However, the presence of *G72* transcript and pLG72 has recurrently been called into question (Benzel et al., 2008). Western blot analysis did not reveal pLG72 expression in primary human astrocytes, glioblastoma (U251), neuroblastoma (SKNAS and SKN-BE), hepatocellular carcinoma (HEP2G), cervical carcinoma (HeLa), and embryonic kidney (293T) cells (Kvajo et al., 2008). Immunocytochemistry revealed pLG72 only in a small fraction (0.1%) of HeLa cells.

Higher *G72* mRNA and pLG72 levels were identified in schizophrenia-affected individuals than in healthy controls (Korostishevsky et al., 2004): a higher pLG72 level was detected in plasma and in the dorsolateral prefrontal cortex of affected individuals than in healthy controls (Korostishevsky et al., 2004; Lin et al., 2014). Interestingly, recent work identified pLG72 protein but not the corresponding mRNA in six brain regions in human *postmortem* samples. In our opinion, the difficult (and sometimes controversial) identification of pLG72 could result from a combination of low recognition efficiency of the commercial antibodies and very low (and strictly controlled) physiological expression levels. This latter hypothesis is also supported by the high level of methylation of CpG sites observed in the *G72* gene in cerebellum (Jagannath et al., 2017a).

The SNP rs2391191 (or M15) in *G72* corresponds to the Arg30Lys substitution in pLG72 (RefSeq NM_172370). This SNP has been linked with lower width of the brain cortex in schizophrenia-affected individuals (Schultz et al., 2011) and with alteration in memory function (Donohoe et al., 2007). *G72* gene was also correlated with other phenotypes and psychiatric disorders, e.g., bipolar disorder and major depression.

The main role of pLG72 is related to its ability to bind the enzyme D-amino acid oxidase (DAAO, EC 1.4.3.3) (Chumakov et al., 2002), i.e., the peroxisomal flavoenzyme attributed to the catabolism of D-serine (Pollegioni and Sacchi, 2010; Sacchi et al., 2012). This D-amino acid is a primary endogenous coagonist of the N-methyl-D-aspartate type glutamate receptor (NMDAR) involved in main functions such as synaptic plasticity, learning, memory, and excitotoxicity (Bear and Malenka, 1994; Henneberger et al., 2013; Paoletti et al., 2013). A decrease in D-serine concentration has been reported in psychiatric diseases such as schizophrenia and bipolar disorder: association studies have linked DAAO and pLG72 with schizophrenia susceptibility. An increase in the level of this neuromodulator has been related to acute and chronic degenerative disorders such as amyotrophic lateral sclerosis (here, the R199W substitution in DAAO has been associated with a familial case; Mitchell et al., 2010), Parkinson's and Alzheimer's diseases. For a review, see (Sacchi et al., 2012, 2016). The opportunity to regulate DAAO activity open the way to modulate D-serine level *in vivo* and thus to act on different pathological states involving alteration in NMDAR function. Immunoprecipitation of human DAAO (hDAAO) from human cortex extracts identified both the band corresponding to hDAAO (≈40 kDa) and the band at 26 kDa corresponding to pLG72: the same results were apparent using pLG72 coimmunoprecipitation (Sacchi et al., 2008). Double immunostaining experiments in brain slices demonstrated that both hDAAO and pLG72 are distributed in the astrocytes, concentrated mostly in the cell bodies, with no signal detected in the processes of astrocytes. This issue will be discussed in depth in the further course of this review.

Over the years, a second role was also proposed for pLG72. Actually, the work of Kvajo et al. (2008) provided convergent lines of evidence that pLG72 is located in mitochondria (see below) and that its overexpression stimulates strong mitochondrial fragmentation. In COS-7, human glioblastoma U251, and human astrocytes cells overexpressing pLG72 (both the wild-type and the R30K variant, which is also targeted to mitochondria, see below), the vesicular distribution of mitochondria was altered and, over time, mitochondria became more fragmented, with tubular forms less prominent (Kvajo et al., 2008). The observed phenomenon might arise from the high, nonphysiological levels of pLG72. Remarkably, the pLG72-induced mitochondrial fragmentation was not followed by increased cell death, but rather was accompanied by an increase in dendritic complexity. After 20 h from transfection, branching points in neurons increased as did the number of dendritic segments and dendritic terminals, with no alteration in the total length of the dendritic tree. The pLG72-induced fragmentation increased the number of shorter mitochondria that are more mobile and thus could be brought to regions of intense growth faster, enabling the increase of dendritic complexity. Notably, pLG72 overexpression seems to induce dendritic branching only during active dendritogenesis in synaptically immature neurons (Kvajo et al., 2008).

In our experiments, a vesicular distribution of mitochondria in pLG72-transfected cells was never observed (Sacchi et al., 2008, 2011; Cappelletti et al., 2014). In the two laboratories different chimeric pLG72 proteins were used for these experiments: in both cases the tag was added at the C-terminus of pLG72 (the putative mitochondrial targeting sequence is located at the N-terminal end) but the myc-tagged pLG72 might have an altered conformation as it was not coimmunoprecipitated with hDAAO from cotransfected COS-7 cells and did not inhibit cellular hDAAO.

Furthermore, a role of pLG72 in the induction of oxidative stress was also proposed by a transcriptomics analysis (Wang et al., 2015). Accordingly, these authors reported an increase in the reactive oxygen species (ROS) by 69% in U87 cells overexpressing pLG72: induction of ROS was quenched by adding a scavenger such as Tempol. Following pLG72 overexpression, the transcriptomics profile identified six genes involved in signal transduction (*RTN1, CNTD2, MAP4K4, HIPK3, TRIM40*, and *SAG*), six genes participating in calcium binding and secondary messenger transduction (*GALNT3, TBC1D8B, SCUBE2, SMOC2, EDNRA*, and *PLA2G5*), and eight genes classified as nucleotide-binding proteins (*HNRNPCL1, FOXR2, ADAL, MAF, UPP1, ARL8A, NR2C1*, and *PSMA1*) (Wang et al., 2015). Concerning the top gene ontology term in biological processes, pLG72 might increase the production of oxidative stress via hydrogen peroxide production and might be involved in nitric oxide biosynthesis/metabolism and in the arginine metabolic process (Wang et al., 2015). A role of pLG72 in oxidative stress was also proposed following the reported interaction with MSRB2, see the section “Additional pLG72 interactors.”

## Biochemical properties of pLG72

In past years, we produced recombinant wild-type, R30K (the SNP associated with schizophrenia susceptibility), and the K62E variant of pLG72 (SNP rs9558562) in *E. coli* cells as inclusion bodies, reaching ≈100 mg/L of fermentation broth (Molla et al., 2006a; Sacchi et al., 2017). In all cases, the recombinant pLG72 proteins were refolded using the anionic detergent N-lauroylsarcosine, a reducing agent and at a basic pH-value. Circular dichroism analyses showed that the refolded pLG72 acquired the tertiary structure and contained a high content of α-helices. Correctly folded pLG72 showed an absorbance maximum centered at 267 nm. The detergent affects the oligomeric state of pLG72: the protein was monodispersed at a detergent concentration ≥0.025%; under optimal conditions, all the pLG72 variants were dimeric (Molla et al., 2006a; Sacchi et al., 2017). The substitutions R30K and K62E only slightly modified the protein conformation, yielding a moderately more rigid conformation than for the previous one (T_m_ was 3–8°C higher than that of wild-type pLG72).

Fluorescence analyses demonstrate that pLG72 possesses a binding site for large, hydrophobic molecules. pLG72 binds aromatic compounds, i.e., the cofactors FAD, FMN, and riboflavin, the antipsychotic drug chlorpromazine (CPZ), the nucleotides GDP/GTPγS, and the sugar β-D-galactopyranoside, see Table 1 (Chumakov et al., 2002; Sacchi et al., 2008, 2017). It was previously proposed that the activity of FMN-containing oxidoreductase of complex I in mitochondria is regulated by pLG72 binding (Otte et al., 2011): as the K_d_ values for FMN/FAD were similar for all pLG72 variants (Sacchi et al., 2017), we exclude that the R30K substitution favors such an interaction. Notwithstanding the ability to bind nucleotides, the pLG72 variants did not show GTPase or ATPase activity (Sacchi et al., 2017).

**Table 1:** Properties of pLG72 variants (Sacchi et al., 2017).

	**pLG72 variants**			
		**R30 (wild-type)**	**R30K**	**K62E**
Binding *K*_d_ (μM)	FAD^a^	54.2 ± 9.3	5.07 ± 3.5	84.4 ± 3.2
	FMN^a^	68.9 ± 1.9	36.4 ± 3.2	50.5 ± 4.5
	CPZ^a^	1.69 ± 0.09	1.56 ± 0.20	1.28 ± 0.03
	GDP^a^	287 ± 22	546 ± 82	690 ± 55
	GTPγS^a^	72.5 ± 14.9	40.0 ± 8.2	64.6 ± 14.8
	hDAAO^b^	3.30 ± 1.00	2.15 ± 0.45	3.40 ± 1.07
		(2.7)^c^ (8.3)^d^		
hDAAO inhibition^e^ (%)		53	15	8
D-/(D+L)-serine^f^ (%)		3.09 ± 1.06	3.52 ± 1.07	3.68 ± 0.97
Half-life (min)		23.9	13.6	21.0

a *Binding was determined following changes in protein fluorescence*.

b *Determined from SPR analysis*.

c *Sacchi et al. (2008)*.

d *Birolo et al. (2016)*.

*^a−d^Standard deviation was calculated from curve fitting*.

e *At 1:2 hDAAO:pLG72 ratio and 30 min of incubation*.

f *Determined by HPLC analysis; standard deviation arises from measurement replicates*.

All pLG72 variants formed the 200-kDa complex with hDAAO with a similar affinity (Table 1). Surface plasmon resonance (SPR) analyses showed a slightly stronger interaction with hDAAO for the R30K protein (K_d_ = 2.2 μM) than for the other pLG72 variants (Sacchi et al., 2017).

## Subcellular localization of pLG72 (and hDAAO)

The subcellular localization of pLG72 was investigated using U87 glioblastoma cells transfected with plasmids encoding for tagged protein variants (Sacchi et al., 2011), demonstrating a mitochondrial localization since:

- The signal for the expressed pLG72–ECFP protein displayed a distinctive “spaghetti like” localization around the nucleus in confocal microscopy.
- In U87 cells stably expressing pLG72–FLAG the signal distribution closely resembled that observed for pLG72–ECFP (their expression was confirmed by Western blot analysis).
- pLG72–ECFP and pLG72–FLAG signals largely colocalized with Mitotracker Green FM dye, a specific mitochondrial marker.
- In U87 cells stably expressing pLG72, a specific signal for this protein was evident in the intact mitochondria following the organelle immunoprecipitation using anti-TOM22 (Translocase of the Outer Mitochondrial Membrane) antibodies.

Our evidence of mitochondrial localization for pLG72 is consistent with that reported for COS-7 transfected cells, glioma U251 and primary neurons (Benzel et al., 2008; Kvajo et al., 2008). The subcellular localization of pLG72 was not modified for the R30K variant, while a diffuse cytoplasmic distribution was observed for pLG72 variants lacking the N-terminal 25 or 35 residues (a partial mitochondrial localization was apparent in ≈40% of the cells; Kvajo et al., 2008). This evidence is in agreement with the classical localization of the mitochondrial targeting sequences at the N-terminal end of the protein. Concerning the subcellular localization of hDAAO, it is known to be a peroxisomal enzyme (Moreno et al., 1999; Sacchi et al., 2008; Pollegioni and Sacchi, 2010). Using cultured human astrocytes, DAAO signal was widely distributed throughout the cytoplasm with a signal pattern resembling that of PMP70, a component of peroxisomal membranes, and a partial localization in the perinuclear region of the cell (Sacchi et al., 2008, 2011). This observation suggests that, in human astrocytes, DAAO is largely confined in the peroxisomes but also partially localized outside these organelles.

The aforementioned experimental evidence implies distinct subcellular localizations for hDAAO and pLG72: their interaction is described in the following paragraph (see subsection in “Cellular studies”).

## hDAAO-pLG72 interaction

### *In vitro* experiments

In 2002, using a yeast two-hybrid system, a clone of hDAAO was isolated from a human brain cDNA library using pLG72 as bait (Chumakov et al., 2002). The specific interaction of pLG72 with hDAAO was confirmed *in vitro* using purified recombinant proteins (Sacchi et al., 2008) by:

- far Western blot analysis under denaturing and native conditions, which is specific for DAAOs from mammalian sources as no signal was observed using the enzyme isolated from yeast;
- quantitative pull-down experiments;
- spectroscopic methods, i.e., by following the fluorescence signal of the hydrophobic probe 8-anilino-naphthalene-1-sulfonate during titration of pLG72 with hDAAO;
- gel-permeation chromatography: a 200-kDa complex made of two pLG72 monomers and two hDAAO homodimers (2 × 18 kDa + 2 × 80 kDa) was produced when the two recombinant proteins were mixed. As a control, the elution volume of yeast DAAO was not affected by adding pLG72. The elution profile was not affected by using the apoprotein form of hDAAO or by adding 40 μM free FAD or 0.1 mM CPZ;
- SPR analysis yielded an apparent K_d_ of ~2–8 μM for the hDAAO–pLG72 complex (Sacchi et al., 2008, 2017; Caldinelli et al., 2010). The presence of 20 μM of the cofactor FAD, an identical concentration of the FAD-competitive inhibitor CPZ, 35 μM of the substrate-competitive inhibitor benzoate, or 50 mM of the physiological substrate D-serine did not affect pLG72 binding and dissociation to immobilized hDAAO (Caldinelli et al., 2010).

Concerning the role of pLG72 binding to hDAAO, at first an increase in enzymatic activity of pig DAAO (80% sequence identity to hDAAO) was reported: accordingly, pLG72 was suggested to activate the flavoenzyme (Chumakov et al., 2002). We replicated the experiment under the same conditions but using our recombinant pLG72 preparation: the interaction of pkDAAO to pLG72 was still apparent in far Western blot experiments but the activity of pkDAAO was decreased (Sacchi et al., 2008). For this reason, we postulated that the key *in vitro* effect of pLG72 binding to hDAAO is its inactivation (Sacchi et al., 2008). Actually, when hDAAO was mixed with increasing amounts of pLG72 (at 0.06% N-lauroylsarcosine), we observed a time-dependent inactivation: such an effect was not due to protein precipitation or proteolysis (Sacchi et al., 2008). The kinetic parameters of hDAAO on the physiological substrate D-serine were not affected by adding pLG72 nor, similarly, was coenzyme binding (Sacchi et al., 2008; Caldinelli et al., 2010). We demonstrated that following a 30-min pre-incubation, pLG72 binding decreases the amount of the flavoenzyme form that is catalytically competent, i.e., the amount of hDAAO-bound FAD cofactor that is reduced by D-serine under anaerobic conditions. The evidence that the same result was also observed using an excess of free FAD excludes that it can be due to the depletion of FAD because of its binding to pLG72.

Limited proteolysis experiments provided additional evidence of an alteration in hDAAO native conformation by pLG72 binding: the half-time for trypsinolysis of the full-length form of hDAAO diminished from 7 to 3.5 min after adding an equimolar amount of pLG72 (Caldinelli et al., 2010). In these conditions, the rate constant for the second phase of holoenzyme degradation was increased, while the time course of proteolysis of the apoprotein form of hDAAO was not altered. (Notably, the hDAAO apoprotein is less compact than the holoenzyme; thus it is more sensitive to proteolysis). Indeed, the FAD cofactor and the substrate analog trifluoro-D-alanine halved the amount of hDAAO degraded in the first (rapid) phase of proteolysis and slowed down the second phase. In contrast, by adding 0.1 mM CPZ the hDAAO-pLG72 complex was fully sensitive to proteolysis, resembling the time course observed for the apoprotein form. Overall, pLG72 binding destabilizes the hDAAO holoenzyme, an effect which is counteracted by the cofactor or by active-site ligands (Caldinelli et al., 2010).

### Cellular studies

The role of pLG72 was investigated at the cellular level in U87 human glioblastoma cells transfected with pEGFP-C3 expression vectors encoding for EGFP, EGFP-hDAAO, or EGFP-pLG72 proteins. D-Serine levels were decreased in cells overexpressing hDAAO, while cotransfection with pLG72 restored D-serine levels and decreased the hDAAO activity (Sacchi et al., 2008). These results highlight the cellular function of hDAAO in D-serine catabolism and confirm the role of pLG72 as a negative effector of the flavoenzyme. For the sake of completeness, we need to mention that pLG72 overexpression reportedly failed to modify the activity of endogenous DAAO in human U251 glioma cells (Kvajo et al., 2008). In this latter experiment, DAAO activity was assayed using D-proline as substrate, a compound which is also oxidized by human D-aspartate oxidase. Indeed, DAAO activity was assayed in SH-SY5Y neuroblastoma cells and in human 1321N1 cells (which have an astrocyte-like phenotype) transfected for hDAAO or cotransfected for hDAAO and pLG72 (Jagannath et al., 2017b). This work reported an increase in hDAAO activity following cotransfection in human kidney HEK293 cells, indicating a cell-specific hDAAO-pLG72 interaction. This is an intriguing proposal that requires further investigation since the DAAO and pLG72 (mRNA and protein) levels differed significantly in the three cell lines used following the nonphysiological overexpression of the two proteins and the effect on cellular D-serine level was not assessed. Indeed, the reported expression of pLG72 in HEK293 cells conflicted with the results of Kvajo et al. (2008).

In order to clarify the interaction between hDAAO and pLG72, U87 pLG72–ECFP cells were transiently transfected with the pEYFP–hDAAO expression vector (Sacchi et al., 2011). The time course of signal localization was investigated by confocal microscopy showing that at 24 h the fluorescence signal of neosynthesized EYFP–hDAAO is spread throughout the cells and is more intense in the perinuclear region where it clearly colocalized with the fluorescence of pLG72–ECFP. At longer times, the signal for EYFP–hDAAO showed a gradual compartmentalization and at 72 h after transfection the typical punctuate distribution of hDAAO was observed (corresponding to the peroxisomal localization) and the colocalization with the signal for pLG72 was marginal. Notably, using the untagged flavoprotein (by using the pIRES–hDAAO vector) the same trend in hDAAO localization was apparent (Sacchi et al., 2011). FRET analysis on U87 pLG72–ECFP cells transiently transfected with the pEYFP–hDAAO plasmid was used to verify the inferred cellular protein–protein interaction: at 24 h upon transfection, the calculated NFRET value in different cells showed a mean figure of 0.436 ± 0.006 supporting the interaction between the two human proteins. These values were appreciably lower at longer times. Thus, we propose that pLG72 and hDAAO could (transiently) interact on the mitochondrial surface: the cytosolic form of the flavoenzyme binds pLG72 before peroxisomes are targeted.

The D-serine level and the D-/L-serine ratio were significantly higher in U87 cells stably expressing pLG72 (5.8 vs. 4.2%) (Sacchi et al., 2011), further supporting the ability of pLG72 to inhibit hDAAO activity (Sacchi et al., 2008). The time course of D-serine concentration, as well as the D-serine concentration at 24 h, differed in U87 control vs. U87 pLG72–ECFP cells following hDAAO transient expression: the interaction of pLG72 to newly synthesized hDAAO, by affecting its catalytic activity, controls the decrease in D-serine concentration. Interestingly, we demonstrated that cytosolic hDAAO is active, having a specific activity similar to that of the peroxisomal form (Sacchi et al., 2011).

At the cellular level, pLG72 affects the half-life of hDAAO. The degradation of hDAAO and pLG72 was investigated in U87 glioblastoma cells stably expressing chimeric proteins with the enhanced yellow fluorescent protein for hDAAO or its cyan variant for pLG72 (Cappelletti et al., 2014). The tag did not alter the biochemical properties of hDAAO, the interaction between the two human proteins, or the cellular distribution of the overexpressed proteins compared to untagged ones. hDAAO is a long-lived protein (t_1/2_ > 60 h) which is degraded by different pathways. The peroxisomal hDAAO was degraded by the lysosomal/endosomal pathway, also termed pexophagy, whereas the cytosolic enzyme fraction was ubiquitinated and targeted to the proteasome. On the other hand, pLG72 is a short-lived protein (t_1/2_ ≈ 25–40 min) and is degraded by the proteasome system, although it was not ubiquitinated (as also confirmed by *in vitro* ubiquitination experiments). Notably, pLG72 cotransfection increased the degradation rate of hDAAO (t_1/2_ from 60 to 6 h), in this way protecting against excessive cellular D-serine depletion. We suggested that pLG72, probably by recruiting additional partners, might target the cytosolic form of hDAAO in the ubiquitin-proteasome system, triggering its degradation (Cappelletti et al., 2014). This could represent a regulation process of D-serine in the hindbrain where DAAO is mainly glial, differently from the forebrain where DAAO is mainly expressed in neurons. Concerning the latter cells, in rat models, the bassoon protein present in the presynaptic zone was reported to interact with DAAO, inhibiting the enzyme and tuning the concentration of D-serine at the synapse, thus affecting NMDAR activity (Popiolek et al., 2011).

**Figure 2:**
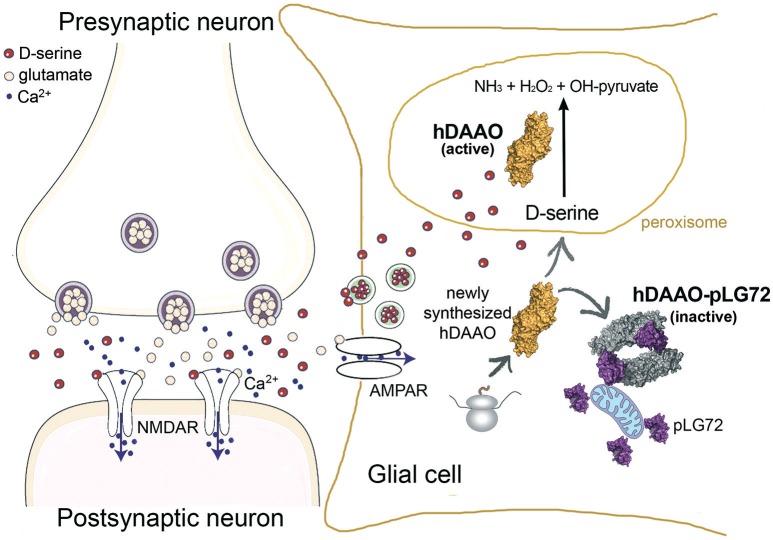
Proposed role of pLG72 on D-serine cellular concentration based on its binding to hDAAO on the cytosolic side of mitochondria in glial cells. An abnormal, lower inhibition of hDAAO activity is due to production of pLG72 variants prone to degradation or with a lower affinity for hDAAO. Hyperactivity of hDAAO generates a nonphysiological decrease in D-serine concentration and, in turn, NMDAR hypofunction.

We can conclude that the interaction of pLG72 to hDAAO on the cytosolic side of the outer mitochondrial membrane induces enzyme inactivation and speeds up its degradation (Figure 2). In this way cytosolic D-serine is preserved and the cells are protected from oxidative stress by hydrogen peroxide generated by the flavoprotein in cytosol.

## pLG72 structure-function relationships

Insight into the structure-function relationships of pLG72 comes from different approaches.

At first, various authors focused on the use of deletion variants, an approach that resulted in differing conclusions (Table 2). By using a yeast two-hybrid approach combined with an assay of DAAO activity, it was reported that the 123–153 and 138–153 regions of pLG72 are involved in DAAO interaction and in enhancing its activity (Chang et al., 2014). A number of C-terminally truncated fragments of pLG72 (namely, 1–20, 1–35, 1–60, 1–122, 1–137, and 95–153) were coexpressed with DAAO in AH109 yeast cells. The first five variants were designed to preserve the predicted secondary structure and the 95–153 fragment so as to contain the conserved sequence present in the three *G72* isoforms. Yeast two-hybrid experiments showed that the 1–137 form does not interact with DAAO while the residues spanning the 138–153 region may interact with the flavoprotein. Actually, pull-down experiments using the 123–153 or 138–153 fragments were 1.4- and 2.2-fold more efficient than the control, respectively, in DAAO immunoprecipitation (Chang et al., 2014). The same peptides also increased the initial rate of DAAO activity (≈1.2-fold vs. control peptides), while full-length pLG72 gave a 3.5-fold increase (Table 2).

**Table 2:** Results gathered on pLG72 by employing deletion variants.

**Variant**	**Region**	**Method**	**Properties**				**References**
			***K*_d, hDAAO_ (μM)**	**T_m_ (°C)**	**Residual hDAAO activity^a^ (%)**	**Comments**	
Wild-type (1–153)	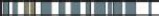	RE	2.66	59.2	16	Mitochondrial; binds hDAAO with no change in kinetic parameters	Sacchi et al., 2008; Birolo et al., 2016
1–64	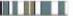	RE	2.2	73.7	72	Compared to the wild-type, the theoretical buried solvent accessible area following hDAAO binding is halved	Birolo et al., 2016
1–94	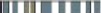		2.1	50.9	62		
1–123	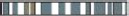		0.4	66.3	47		
72–153	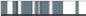		3.7	64.6	50		
122–153	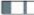	PS			110	Unspecific binding	
26–153	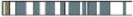	RC				Partial loss of mitochondrial localization	Kvajo et al., 2008
36–153	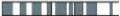					Partial loss of mitochondrial localization	
51–153	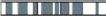						
1–110	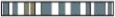					Low expression yield (probably because of protein instability)	
1–120	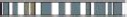						
1–20	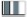	Y2				No interaction with hDAAO	Chang et al., 2014
1–35	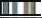						
1–60	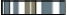						
1–122	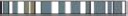						
1–137	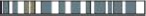						
95–153	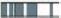					Interacts with hDAAO	
123–153	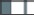					Interacts with hDAAO; 1.4-fold more efficient in hDAAO coimmunoprecipitation than wild-type; increases hDAAO activity (20–30%)^b^	
138–153	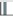					Interacts with hDAAO; 2.2-fold more efficient in hDAAO coimmunoprecipitation than wild-type; increases hDAAO activity	
1–71	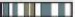	MM				Similar to membrane bound proteins (e.g., membrane transporters or O-GlcNAc transferase)	Kato and Fukui, 2017
72–153	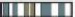					Similar to adaptor proteins (type I DNA methyl transferase, RH bundle subdomain of G protein-coupled receptor kinase)	

*RE, characterization of pure recombinant protein expressed in E. coli; RC, overproduction in COS-7 cells; Y2, yeast two-hybrid system; PS, peptide synthesis; MM, molecular modeling*.

a *At 1:2 hDAAO:pLG72 ratio and 30 min of incubation*.

b *3.5-fold increase in hDAAO activity with wild-type pLG72*.

**Figure 3:**
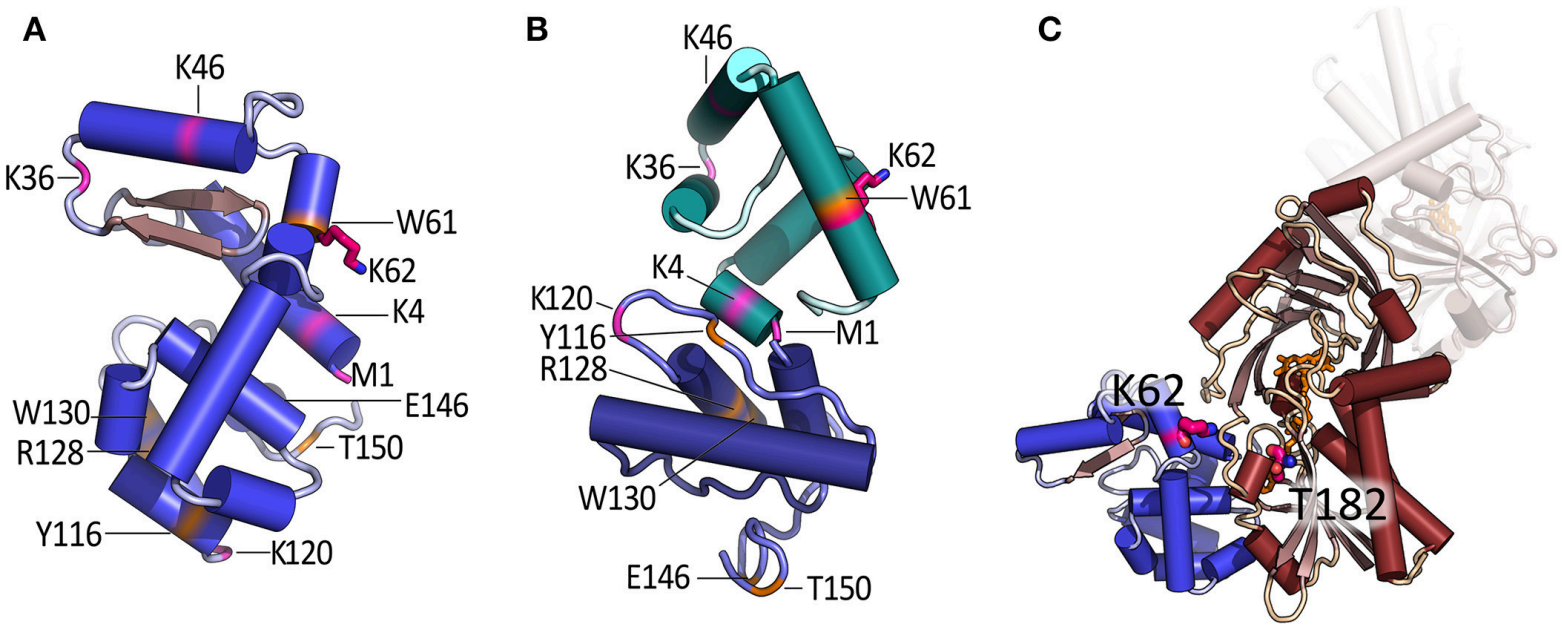
Model of the tertiary structure of pLG72 and proposed mode of interaction with hDAAO. **(A)** Homology 3D model of pLG72 produced by I-TASSER server, i.e., pLG72-VA (Birolo et al., 2016) and **(B)** model produced by hybrid *ab initio* approach (Kato and Fukui, 2017). Proteolytic sites are shown in orange, residues modified by BS^3^ cross-linking reagent are shown in pink, and cross-linked K62 residue is shown in purple. **(C)** Model of hDAAO–pLG72 complex as predicted by ZDOCK server; pLG72 model is in blue, hDAAO dimer in brown, and cross-linked K62 (pLG72), and T182 (hDAAO) are shown in purple (Birolo et al., 2016). For details see Figure 1.

We generated the following pLG72 variants in *E. coli* cells: 1–123 (lacking the putative disordered C-terminal portion), 1–94, 72–153 (the region recognized by the Pfam database as an interaction domain), and 1–64 (Birolo et al., 2016). Interaction analysis by SPR using hDAAO immobilized on the chip surface demonstrated that all pLG72 deletion variants interacted with the flavoenzyme with a K_d_ in the micromolar range: deletion of the C-terminal portion favored the flavoenzyme binding (the 1–123 variant bound to hDAAO with a higher affinity in comparison to the full-length pLG72) while the lack of the N-terminal region in the 72–153 variant slightly negatively affected the hDAAO–pLG72 interaction and the 122–153 polypeptide did bind aspecifically to hDAAO (Table 2). *In silico* protein-protein interaction studies indicated that specific residues belonging to loops in the 1–92 region of pLG72 might interact with hDAAO (Figure 3, see below): actually, the strongest decrease in affinity for hDAAO binding was observed for the 72–153 deleted form of pLG72 lacking the residue K62 identified by cross-linking experiments (see below). On the other hand, only the last three residues in the C-terminal part of pLG72 seem to strictly interact with hDAAO, in agreement with the moderate alterations in binding affinity determined for the variants lacking the C-terminal region. Following the deletion of the region encompassing residues 65–153, the buried solvent-accessible area in the complex was proposed to be halved in comparison to the full-length protein (520 Å^2^).

Employing pLG72 at 2-fold molar excess vs. hDAAO, all the deletion variants produced by Birolo et al. (2016) less effectively impacted hDAAO activity than the full-length pLG72: 25–50% of inhibition vs. >80% (Table 2). The C-terminal portion of pLG72 seems to be involved in the changes in hDAAO conformation associated with abolition of its activity. The lowest inhibition of hDAAO activity was observed for the shorter N-terminal pLG72 forms (i.e., 1–64 and 1–94). Notably, the results gathered on the 122–153 pLG72 fragment differ from previous studies reporting a stronger interaction with hDAAO as compared to the full-length pLG72 (Chang et al., 2014). The discrepancy can be attributed to the experimental methods used: our result arises from gel-permeation chromatography and SPR experiments on the native protein while the previous evidence is founded on a pull-down experiment employing a biotinylated pLG72 form. The reported increase in hDAAO activity at increasing pLG72 concentrations represents a further discrepancy (Chang et al., 2014), which can be rationalized based on the absence of exogenous FAD in the hDAAO assay generating a large amount of the inactive apoprotein form (FAD is mandatory for the assay mixture because of the weak interaction with hDAAO apoprotein) (Molla et al., 2006b).

Deletion of the N-terminal 25 amino acids partially abolished mitochondrial targeting: only ~ 40% of pLG72 was still localized to mitochondria, the main signal resulted in a diffuse cytoplasmic distribution (Kvajo et al., 2008). The pLG72 variant lacking the N-terminal 35 amino acids still showed a partial mitochondrial localization, whereas the absence of 50 residues at the N-terminus prompted protein degradation. On the other hand, deletion of 33 or 43 amino acids at the C-terminal end did not abolish its mitochondrial localization but affected the expression level, probably by altering the protein stability (Kvajo et al., 2008).

Notably, we cannot exclude a conformational change in comparison with the tertiary structure of the wild-type protein in any of the experiments based on pLG72 deletion variants.

The hDAAO–pLG72 complex was investigated at low resolution by limited proteolysis using different proteases coupled to ESI/MS analyses and a large excess of pLG72 protein (Birolo et al., 2016). These experiments further confirmed that pLG72 binding induces a conformational change of hDAAO, which becomes more accessible to proteases in the complex. In detail, the peptide bonds following L189, R191, L194, E249, and L250 were the main site of proteolysis in hDAAO alone but were not recognized in the pLG72-complexed form. In the latter, the peptide bonds following Y34, Y55, F167, N180, L296, R297, and E336 were proteolytically hydrolyzed. The increased sensitivity of hDAAO to proteolysis in the complex with pLG72 (Caldinelli et al., 2010; Birolo et al., 2016) implies that the overall compactness of hDAAO is decreased by this interaction. Limited proteolysis experiments indicate that conformational changes occurred in hDAAO at the C-terminal portion, which became less flexible and exposed, and at the N-terminal portion, which was only cleavable in the presence of pLG72. A more stable protein (T_m_ is 7°C higher) is obtained after deleting the C-terminal end of pLG72, i.e., the region with the highest degree of disorder (Table 2). Indeed, cross-linking experiments using bis(sulfosuccinimidyl)suberate (BS^3^) identified one cross-link between hDAAO and pLG72, i.e., K62 of pLG72 and T182 of hDAAO.

A further approach was based on structure modeling. A preliminary attempt resulted in the production of a tentative model of pLG72 structure predicted by MODELLER 9v10 (Sehgal et al., 2013). Unfortunately, the reliability of this model is quite low due to the lack of suitable templates, undermining the conclusions the authors made based on subsequent computational analyses. This pLG72 model was based on Gα12 protein (PDB code 1ZCA). This protein shares an overall sequence identity of ~10% with pLG72 and, even considering only a part of the template (~30%), the sequence identity was less than 30% with an extremely high BLAST E-value (4.9). Using this preliminary pLG72 model, fragments 123–153 (consisting of loop-helix-loop conformation) and 138–153 (a hairpin loop) were extracted from the predicted structure and docked to DAAO (Chang et al., 2014). Residues R38, K163, W185, G187, A188, R191, P193–Q196, and W247–L250 in hDAAO were suggested to interact with both pLG72 peptides: these hDAAO residues belong to four loops located on the protein surface. Later on, the same authors suggested that residues within the C-terminal end of pLG72 play a crucial role in ligand binding (Sehgal et al., 2015).

Most recently, reliable homology models of pLG72 tertiary structure were independently predicted by two research groups. Our group developed a three-dimensional structure model of pLG72 (named pLG72-VA) by using homology modeling and the I-TASSER server (Figure 3A) (Birolo et al., 2016). Its reliability was evaluated considering the location of the exposed regions identified by limited proteolysis, the position of K62 (the residue involved in cross-linking with hDAAO), the α-helix content (≈62%, a value in agreement with the experimental figure of 73% determined by CD analysis), and the quality level by the SAVES metaserver.

Using the pLG72-VA model, *in silico* molecular docking analysis identified the site and the mode of CPZ binding: the predicted dissociation constant was close to the experimental value (Table 1; Sacchi et al., 2008; Caldinelli et al., 2010). Next, the hDAAO-pLG72 mode of interaction was predicted *in silico* by protein–protein molecular docking using the ZDOCK server, which evaluated all potential pLG72-hDAAO complexes using an energy-based scoring function. Potential complexes were filtered so that the distance between the side chain T182-OH of hDAAO and the ε-NH_2_ group of K62 of pLG72 was within the distance constraint for the BS3 cross-linker (12.2 vs. 11.4 Å, respectively, Figure 3C). Three alternative complexes were generated and the one showing the highest agreement with limited proteolysis results and the widest hDAAO-pLG72 surface of contact was taken into consideration. Actually, 1038 Å^2^ in hDAAO and 1072 Å^2^ in pLG72 are excluded from solvent contact in the selected hDAAO–pLG72 complex, pointing to a stable interaction (Birolo et al., 2016).

In 2017, an alternative model of the 3D structure of pLG72 was produced using a hybrid *ab initio* approach in which the region 1–71 and 72–153 of pLG72 were independently modeled (Kato and Fukui, 2017). Comparison of the pLG72 model by Kato and Fukui (2017) with our model (Birolo et al., 2016) reveals a general similarity in the overall molecular shape and α-helix content. In addition, the position of the key residue K62 is similar between the two models, although the orientation is different (Figures 3A,B). Interestingly, the predicted structure of the N-terminal region was similar to that of membrane-bound proteins, in agreement with the proposed location of pLG72 on the surface of membranes of mitochondria (Kvajo et al., 2008; Sacchi et al., 2011). The N-terminal domain might bind to the phosphate groups of membrane lipids. On the other hand, the C-terminal region structurally resembled adaptor proteins, in particular the helix bundle that serves as the dimer interface of modification subunit of type I DNA methyltransferase. Accordingly, these authors suggested that pLG72 serves as an adaptor protein (Kato and Fukui, 2017).

## The R30K substitution

The introduction of the conservative R30K substitution in pLG72 slightly altered the protein conformation: the main effect was a more rigid conformation (Sacchi et al., 2017). This residue was proposed to line the binding pocket for hydrophobic ligands (Birolo et al., 2016). Concerning the hDAAO inhibition after 30 min of incubation, the R30 variant was less effective, while the K62E pLG72 resulted in the highest level of inhibition (Table 1). The latter result correlates well with the highest increase in D-/(D+L)-serine level observed in U87 cells stably overexpressing the pLG72 variants as compared to the R30 pLG72 (Table 1). Concerning the stronger inhibition of hDAAO observed for the non-conservative K62E substitution, it can be generated by an additional charge-dipole interaction between E62 (a residue proposed to be located on the protein surface) in the pLG72 variant and T182 in hDAAO, the two interacting residues identified by cross-linking experiments (Birolo et al., 2016).

As the half-life of pLG72 R30K variant is significantly lower than that of its R30 variant (half-life of 13.6 vs. 23.9 min, Table 1), a value which is not modified by the K62E substitution, it is less effective in inactivating hDAAO under physiological conditions, thus producing an excessive decrease in D-serine cellular level (Figure 2), a change that can result in neurotransmission alterations since it promotes NMDAR hypofunction and schizophrenia onset (Lisman et al., 2008).

## Additional pLG72 interactors

Searching for a binding partner of pLG72 by a yeast two-hybrid screen, the enzyme methionine-R-sulfoxide reductase B2 (MSRB2, a mitochondrial protein) was identified (Otte et al., 2014). By using a modified version of the LUMIER method to detect protein–protein interactions in HEK-293 cells, the MSRB2-pLG72 interaction was further verified. The result did not change when using GFP or myc-tagged chimeric pLG72 proteins. In neuroblastoma N2a cells, the signals for MSRB2-GFP and pLG72-GFP showed a large colocalization in cotransfected cells, and their signal also colocalized with the one for the mitochondria-specific marker Mitotracker.

The same authors failed to confirm the interaction of pLG72 with the DAAO enzyme but, intriguingly, proposed that pLG72 was involved in the cellular oxidative stress defense system. Actually, MSRB2 catalyzes the conversion of the methionine R-sulfoxide to the reduced form of methionine: its silencing exacerbates the sensitivity toward oxidative stress, while its overexpression protects from oxidative stress (Otte et al., 2014). Changes in pLG72 expression level can increase mitochondrial oxidative stress and damage, by altering MSRB2 function. Notably, NMDAR were also identified on the inner membrane of mitochondria (Korde and Maragos, 2012): these receptors, which are insensitive to glycine as coagonist, might represent a further conduit for calcium entry in mitochondria.

Indeed, pLG72 was proposed to interact with the mitochondrial FMN-containing oxidoreductase of complex I, see above (Otte et al., 2011).

## pLG72 and hDAAO inhibition

A number of research groups worldwide focused on the identification/production of hDAAO inhibitors as a way to modulate NMDAR function acting on D-serine, reviewed in Sacchi et al. (2013). As pLG72 is supposed to modulate hDAAO activity *in vivo*, we recently focused on designing a high-throughput screen method to identify hDAAO inhibitors sensitive to the presence of pLG72 (Terry-Lorenzo et al., 2015). Two classes of compounds sensitive to pLG72 were identified: class A compounds were more potent when pLG72 was present. It is disappointing that the structure of these molecules is unknown and the mechanism of inhibition was not investigated further. The same work identified class C hDAAO inhibitors that are less potent in the presence of pLG72. These latter compounds showed a novel mechanism of hDAAO inhibition as they acted via a mixed-inhibition mechanism of action with respect to both D-serine and FAD. These compounds showed an undetectable, very slow off-rate: the prototypical compound 2 interacted covalently with all five hDAAO cysteines, probably because of the large amount of hDAAO present in the apoprotein (weakly packed) form in the absence of excess FAD. We proposed that in the protein-protein complex, pLG72 partially shields the cysteines of hDAAO from class C compound access, either directly or by altering the protein conformation. This investigation also confirmed that the primary effect of pLG72 was hDAAO inhibition and highlighted the specificity of class C compounds as they did not inhibit glucose oxidase or D-aspartate oxidase (Terry-Lorenzo et al., 2015).

## Conclusions

The role of pLG72 in human brain represents a puzzling and relevant subject. Up to now, the reported investigations reached conflicting conclusions, thus leaving a number of open issues. A main goal of future investigations will be to clarify the relationships between pLG72 expression, mitochondria fragmentation and oxidative stress, also seeking for a link with the inhibitory effect of hDAAO activity. This is also strictly connected with the identification of additional binding partners and their role on pLG72 subcellular localization and half-life.

In order to fully understand the pLG72-hDAAO interaction the pLG72 three-dimensional structure needs to be resolved to give more detailed knowledge of its structure–function relationships. There is an urgent need for novel drugs to treat schizophrenia, and thus the definition of the details concerning the residues involved in the protein-protein interaction will allow us to design molecules to modulate such an interaction and thus D-serine cellular levels acting on hDAAO function. The role of epigenetic modifications on *G72* and *DAAO* genes expression in different cell lines during development and under pathological conditions will help clarify the link with main human brain functions. Actually, genetic analyses did not reach a conclusion concerning association of the *G72* gene with schizophrenia susceptibility and other psychiatric disorders (Sacchi et al., 2016). Anyway, functional magnetic resonance imaging analyses showed that the SNP resulting in R30K substitution in pLG72 was related to increase cortical thickness in schizophrenia but not in healthy controls (Schultz et al., 2011): this SNP was also proposed as a novel marker of alterations in memory performance in schizophrenia-affected individuals. On this side, the level of pLG72 protein expression in schizophrenic patients vs. healthy controls is still under debate. Similarly, an open issue is related to the detection of pLG72 (protein and mRNA) in different brain regions: the present results reached conflicting conclusions. The production of sensitive and selective anti-pLG72 antibodies is mandatory to accurately detect the human protein in brain samples (see Introduction section).

## Author contributions

All authors listed have made a substantial, direct and intellectual contribution to the work, and approved it for publication.

### Conflict of interest statement

The authors declare that the research was conducted in the absence of any commercial or financial relationships that could be construed as a potential conflict of interest.

## Abbreviations

BS^3^: bis(sulfosuccinimidyl)suberate
CPZ: chloropromazine
DAAO: D-amino acid oxidase
hDAAO: human DAAO
NMDAR: N-methyl-D-aspartate type glutamate receptor
SNP: single nucleotide polymorphism
SPR: surface plasmon resonance.
